# Evaluation of the *PLAC8* Gene in Mexican Women With and Without Preeclampsia and Obesity

**DOI:** 10.3389/fmed.2022.795309

**Published:** 2022-02-17

**Authors:** Laura Jazel Barragán-Zúñiga, Laurence A. Marchat, Ivo Carrasco-Wong, Ricardo Blanco-Castaneda, José M. Salas-Pacheco, Luis Ernesto Simental-Mendia, Miguel Mauricio Correa-Ramírez, Martha Sosa-Macías, Jaime Gutiérrez, Carlos Galaviz-Hernandez

**Affiliations:** ^1^Laboratorio de Biología Molecular, Academia De Genómica, Instituto Politécnico Nacional-Centro Interdisciplinario de Investigacira el Desarrollo Integral Regional Durango, Durango, Mexico; ^2^Red Iberoamericana de Alteraciones Vasculares en Trastornos del Embarazo, Chillán, Chile; ^3^Laboratorio de Biomedicina Molecular II, Escuela Nacional de Medicina y Homeopatía, Instituto Politécnico Nacional, Mexico City, Mexico; ^4^Cellular Signaling and Differentiation Laboratory, School of Medical Technology, Health Sciences Faculty, Universidad San Sebastian, Santiago, Chile; ^5^Laboratorio de Biología Molecular, Instituto de Investigación Científica, Universidad Juárez del Estado de Durango, Durango, Mexico; ^6^Biomedical Research Unit, Mexican Social Security Institute, Durango, Mexico; ^7^Laboratorio de Biología Molecular, Academia de Entomología, Instituto Politécnico Nacional-Centro Interdisciplinario de Investigacira el Desarrollo Integral Regional Durango, Durango, Mexico

**Keywords:** preeclampsia, obesity, placenta, *PLAC8*, gene expression, protein expression, nucleotide variations

## Abstract

Preeclampsia (PE) is a leading cause of maternal-fetal mortality worldwide, and obesity is an important risk factor. Genes associated with pathophysiological events common to preeclampsia and obesity, such as *PLAC8*, remain to be studied; therefore, the aim of the present study was to evaluate this gene in the placentas of women affected with preeclampsia and healthy pregnant women. This case-controlled study included 71 healthy and 64 preeclampsia pregnancies. Gene expression was evaluated in primary human cytotrophoblasts (PHCT) from six normal and six preeclampsia pregnancies, and protein expression was verified in placentas from five healthy and six preeclampsia pregnancies. The whole coding and 5′ regions of the *PLAC8* gene were sequenced from healthy (*n* = 10) and preeclamptic (*n* = 10) pregnancies. The presence of the observed nucleotide variations was analyzed by RT-PCR in the total population. Statistical analyses were performed accordingly. Obesity was associated with severe preeclampsia (SPE) (OR = 3.34; CI 95% 1.3–8.2, *p* < 0.01). Significantly higher mRNA and protein expression was observed in preeclamptic vs. healthy placentas (*p* < 0.05). After sequencing, a single nucleotide variation was identified in 10 cases and one control (*p* < 0.01), which was then evaluated in the total population showing no association with preeclampsia. This preliminary study confirms the association of SPE with obesity and suggests higher expression of *PLAC8* mRNA and protein in placentas from preeclampsia. No differences in nucleotide variations between cases and controls of the whole population were observed. Further research is required to evaluate the implications of higher gene/protein expression in preeclampsia and the causes of such variation.

## Introduction

Preeclampsia represents the most frequent hypertensive disorder of pregnancy (2–8% globally) associated with maternal-fetal complications (1). Preeclampsia is characterized by the onset of hypertension after 20 weeks of gestation and proteinuria >300 mg in 24 h (2). Depending on its severity, the condition is classified as mild preeclampsia (MPE) and severe preeclampsia (SPE) (2, 3).

According to the WHO, preeclampsia prevalence in Mexico is estimated to be between 10 and 14%, and it leads to 4,000 maternal deaths annually. The presence of obesity, a body mass index (BMI) ≥30 kg/m^2^, and comorbidities such as type 2 diabetes mellitus (DMT2), represent a great risk for preeclampsia (4). The risk increases three times compared to women with normal weight [odds ratio (OR) 2.9] (5), which has been observed in different populations, such as Americans, Canadians, and New Zealanders (6, 7). In Latin American populations, a linear trend of risk was observed between an increase in BMI and preeclampsia (8).

Despite these correlations, the molecular mechanism underlying the relationships between preeclampsia and obesity is not well-understood. Recently, the transcriptomic evaluation of placentas in women with preeclampsia (9) allowed for the identification of changes in the expression of genes related to obesity, such as leptin (*LEP*) (10), leptin receptor (*LEPR*) (11), adiponectin (*ADIPOQ*) (12), alpha-ketoglutarate dependent dioxygenase (*FTO*) (13), and peroxisome proliferator activated receptor gamma (*PPAR-y*) (14). Another identification strategy has been the evaluation of genes related to physiological processes common to preeclampsia and obesity, including the *PLAC8* gene (placenta-specific 8). The underexpression of the *PLAC8* gene promotes preadipocyte differentiation (15), while its overexpression is associated with failures in the placental implantation process (16). *PLAC8* was initially characterized in the early stages of mouse placental development and is highly conserved to its equivalent ortholog in humans (17). In the latter, *PLAC8* is located on chromosome 4 at position q21.22. Its coding region includes five exons that encode a 12.5 kDa protein (GeneCards^1^). *In silico* evaluation revealed the presence of 6,224 single nucleotide variants (SNVs) in the coding region, without any reported clinical repercussions (https://www.ncbi.nlm.nih.gov/gene/51316). Since most of the studies in the search for nucleotide variations associated with preeclampsia are carried out in affected mothers, the impact of these SNVs or polymorphisms on the fetus and/or the placenta has not been studied. Thus, to evaluate the effects that fetal genetics may have on the development of preeclampsia, the assessment of this tissue is critical as a first step in the identification of new genes and/or proteins involved in the origin and/or development of preeclampsia. Such work could lead us to identify potential markers of this syndrome.

Because the *PLAC8* gene is relevant for placental implantation processes and adipogenesis, the aim of this study was to comparatively evaluate *PLAC8* in the placentas of Mexican women with and without preeclampsia and obesity.

## Methods

### Study Groups

We conducted a case-controlled study, and the eligible participants were pregnant women with and without preeclampsia. The diagnosis of preeclampsia was performed by a gynecologist according to the criteria of the American College of Obstetricians and Gynecologists (ACOG) (2). The exclusion criteria included essential hypertension, altered renal function, twin pregnancy, recurrent miscarriage, and placental abruption. The protocol was approved by the Ethics and Research Committee of the Ministry of Health at Durango City (approval N. 094) in accordance with the Code of Ethics of the Declaration of Helsinki and its later amendments. After signing the informed consent letter, eligible women were invited to participate at the General Hospital of Durango Mexico (from August 2017 to August 2019). Human placentas were obtained from healthy (HP) (*n* = 71) and preeclamptic pregnancies, which were classified as MPE (*n* = 33) and SPE (*n* = 31).

### Measurements

The weight and height of the participants were measured using a fixed scale with a stadimeter in the standing position, wearing only light clothing and without shoes. Body mass index was calculated as weight (kg) divided by height (m) squared.

Blood pressure was measured according to the technique recommended by the Seventh Report of the Joint National Committee on Prevention, Detection, Evaluation, and Treatment of High Blood Pressure (18).

### Definitions

Mild preeclampsia and SPE were classified according to the criteria from the Working Group Report on High Blood Pressure in Pregnancy (2). Proteinuria was identified as ≥300 mg/24 h (19). Normal pregestational weight and obesity were defined as a BMI <25 and ≥30 kg/m^2^, respectively (8).

### Placental Tissue Collection

After the delivery of the placenta (collection time no longer than 30 min), biopsies of 5 × 5 mm were obtained from cotyledons of the maternal side and washed in cold 1x phosphate buffered saline (PBS; mmol/L: 130 NaCl, 2.7 KCl, 0.8 Na_2_HPO_4_, 1.4 KH_2_PO_4_, 4°C, pH 7.4). The sample was then immersed in RNAlater 1:1 (Sigma-Aldrich™) and stored at −80°C for further DNA/RNA extraction.

### Primary Human Cytotrophoblast Isolation

Primary human cytotrophoblast (PHCT) was isolated from fresh placental villus tissue obtained at term from normal (*n* = 6) and preeclampsia (*n* = 6) pregnancies, as we previously described (20). After the serial enzymatic digestion steps of the tissue, PHCT was isolated from gradient fractions between 35 and 55% in a Percoll gradient (10–70%) (GE Healthcare, USA). The purified PHCT was washed three times with PBS and immediately homogenized to obtain RNA extracts. The purity of the PHCT preparation was determined to be 93–99% by staining the cells with the specific markers anti-cytokeratin 7 (CK7), anti-vimentin, anti-E-cadherin, and anti-von Willebrand factor (vWF) (Novus Biologicals, USA), followed by flow cytometry analysis in FACSDiva (BD Biosciences, USA), as previously described (20).

### RNA Extraction From PHCT, Synthesis of cDNA, and Studies of Gene Expression

Total RNA was isolated from PHCT cell pellets using a total RNA Isolation Kit according to the instructions of the manufacturer (Life Technologies, USA). Reverse transcription into complementary DNA was performed using SuperScript® III Reverse Transcriptase and random hexamers according to the instructions of the manufacturer (Life Technologies, USA).

The cDNA from PHCT from normal term (*n* = 6) or preeclampsia pregnancy (*n* = 6) was analyzed by real-time RT-PCR performed in a Step-One system (Applied Biosystems, Thermo Scientific, USA) using Takyon™ Rox SYBR® QPCR master mix (Eurogenet, BE.). GAPDH was used for normalization to quantify relative mRNA expression levels, and relative changes in mRNA expression were calculated using the comparative cycle method (2-^ΔΔCt^). Real-time PCR primer sequences were as follows: *PLAC8-forward* (5′-GTCGCAATGAGGACTCTCTAC-3′), *PLAC8-reverse* (5′-CAATGAGGACAGCAAAGAGTT-3′), *GAPDH-forward* (5′-AGGTCGGTGTGAACGGATTTG-3′), and *GAPDH-reverse* (5′-TGTAGACCATGTAGTTGAGGTCA-3) (IDT DNA technologies, Coralville, IA, USA).

### Protein Expression Studies

For the western blot assay, protein extracts of placental tissue (term of gestation) from HP (*n* = 5) and SPE (*n* = 6) were obtained from 100 mg of tissue homogenized in 1 ml of EDTA/Tris-HCl solution (10 mmol/L EDTA, 50 mmol/L Tris-HCl, pH 8.3). Further homogenization was carried out with 1 ml of SDS/glycerol/Tris solution (4% SDS, 20% glycerol, 125 mmol/L Tris/HCl, pH 6.8) and sonication (four cycles, 10 s, 100 W, 4°C). The concentration of total protein was determined with bicinchoninic acid with a Protein Assay Kit (micro-BCA) (Thermo-Fisher Scientific) according to the instructions of the manufacturer. Total protein extracts were adjusted to 50 μg, separated by polyacrylamide gel (15%) electrophoresis (SDS-PAGE) and transferred to P polyvinylidene difluoride membranes (Bio-Rad, USA). The membranes were incubated for 1 h with Tris-buffered saline-Tween 20 (TBS-T) containing 5% non-fat dry milk. Proteins were probed with primary polyclonal rabbit anti-PLAC8 (1:500 dilution, 18 h, 4°C) (Abcam ab122652) and monoclonal rabbit anti-β-actin (1:5,000 dilution, 18 h, 4°C) (Sigma-Aldrich) in TBS-T 5% non-fat dry milk; β-actin was used as a loading control. The membranes were incubated (1 h) with goat anti-rabbit antibody (Thermo Scientific, USA). Proteins were detected by enhanced chemiluminescence in an ImageQuant LAS 500 chemiluminescence CCD camera (CYTIVA, USA) and quantified by densitometry.

### DNA Extraction and Genotyping Analysis

DNA was extracted from placental tissue using the Miller technique (21) with slight modifications. Briefly, the DNA integrity and concentration were verified through 1% agarose gel electrophoresis and spectrophotometry in a NanoDrop 2000™ (Thermo Fisher™), respectively. Sequencing was carried out in the five exons and the potential regulatory regions surrounding the transcription start site (TSS) of the *PLAC8* gene (Genome Browser, https://genome.ucsc.edu/cgi-bin/hgGateway). Specific oligonucleotides were designed using Primer3 software and verified on the NCBI platform (Table 1). The sequences in randomly selected samples from 10 cases and 10 controls were evaluated by Sanger sequencing with BigDye 3.1™ in an ABI Prism-310™ Genetic Analyzer sequencer from Applied Biosystems™. A visual inspection and comparisons with NCBI databases using BLAST software were carried out to search for changes in the sequence of all coding regions of each selected sample. The potential identified variables of interest were sought in the whole population through real-time PCR using specific TaqMan probes in a StepOne Thermocycler™ (Applied Biosystems™).

**Table 1:** Oligonucleotide sequences for exon and promoter amplification of the *PLAC8* gene.

**Exon**	**Oligonucleotides**	**Sequence**
E1	E1 forward	5′-cagggacaggtctgaagca-3′
	E1 reverse	5′-aggtctggtcccagcagag-3′
E2	E2 forward	5′-gaatgggggaaggattgagt-3′
	E2 reverse	5′-ccactctgccttttcagtcc-3′
E3	E3 forward	5′-tggggtgcatatttgagtga-3′
	E3 reverse	5′-attttgtttcccgtgccttg-3′
E4	E4 forward	5′-gaagaaaattaaagccaatcttga-3′
	E4 reverse	5′-ggcaactctttgctgtcctc-3′
	E4 overlapped forward	5′-gaaagtacgcatggctctcc-3′
	E4 overlapped reverse	5′-tgccttctgcttctcttcaa-3′
E5	E5 forward	5′-ggcaaaagtgttggctaaatg-3′
	E5 reverse	5′-ctcccaaggtgctggaatta-3′
	E5 overlapped forward (1)	5′-ccaaaatctcctgctaagaaacc-3′
	E5 overlapped reverse (1)	5′-tccacctcctggatacaagc-3′
	E5 overlapped forward (2)	5′-atcccagctactcgggagac-3′
	E5 overlapped reverse (2)	5′-tgaatgttgtccctgaacttagc-3′
	E5 overlapped forward (3)	5′-aagtttataagccaggaaattcg-3′
	E5 overlapped reverse (3)	5′-ttgggtgccagatacatgag-3′
**TSS** ^**‡**^		
1	P1forward	5′-aaccacttggactgctcttt-3′
	P1 reverse	5′-ggcactcaataaaatatttgctgaat-3′
2	P3 forward	5′-gagactccacctcaacaaaacc-3′
	P3 reverse	5′-ctaccacacagcagggtcc-3′

‡ *Transcription start site surrounding region*.

### Statistical Analysis

Normally distributed variables are expressed as the mean values ± SD. Categorical variables are expressed as *N* (number) with percentage (%). Comparisons between two groups were performed by Student's *t*-test for continuous variables and χ^2^ analyses for categorical variables. The correlations between preeclampsia and other parameters were analyzed by Pearson's coefficient. Genotype frequencies were obtained by direct count, allele frequencies were calculated, and Hardy-Weinberg equilibrium (HWE) was obtained through a χ^2^-test. Data were analyzed using IBM SPSS statistical software (version 24.0; SPSS Inc., Chicago, IL, USA), GraphPad Prism software (version 7.0; CA, USA), and SNP Stats (http://bioinfo.iconcologia.net/SNPstats). Statistically significant differences were considered at *p* < 0.05.

## Results

The characteristics of 71 HP controls, 33 women with MPE, and 31 women with SPE are shown in Table 2. Reproductive and lifestyle characteristics of the cases and controls in the study showed no differences. However, T2DM was identified in four cases (two women in MPE and two in SPE) but not in the controls, with differences observed between the cases and controls (*p* = 0.001) (OR = 2.18; CI 95% 1.8–2.6, *p* < 0.04). The frequency of obesity (BMI ≥30 kg/m^2^) was significantly higher in the SPE group than in the control group (*p* = 0.015). Multivariate regression analysis showed a significant association between obesity and SPE (OR = 3.34; CI 95% 1.3–8.2, *p* < 0.01). Significant differences in other variables were not observed between the study groups.

**Table 2:** Medical, reproductive, and lifestyle characteristics of the participants according to preeclampsia classification.

	**Total (*n)***	**Control *n* (%)**	**Mild *n* (%)**	**Severe *n* (%)**	***P***
**Age**					
>35 years	18	9 (50)	3 (16.7)	6 (33.3)	0.412
<35 years	114	62 (54.4)	29 (25.4)	23 (20.2)	
**Blood pressure**					
Systolic pressure (mmHg)	135	111.48 ± 8.6	146.34 ± 9.6	179.34 ± 13.9	0.001^*^
Diastolic pressure (mmHg)	135	72 ± 7.3	94.71 ± 11.8	108.29 ± 14.9	0.001^*^
**Proteinuria**	64	0 (0)	31 (48.43)	33 (51.56)	0.05^*^
300 mg/24 h	1 (1.56)		0 (0)	1 (1.56)	
1–2 g/L 24 h	6 (9.37)		1 (1.56)	5 (7.81)	
2–3 g/L 24 h	55 (85.93)		29 (45.31)	26 (40.62)	
>3 g/L 24 h	2 (3.12)		1 (1.56)	1 (1.56)	
**BMI**					
>30	65	30 (46.2)	13 (20)	22 (33.8)	0.015^*^
<30	70	41(58.6)	20 (28.6)	9 (12.9)	
**Personal history of preeclampsia**	12	4 (33.3)	3 (25)	5 (41.7)	0.185
**Personal history of T2DM**	4	0 (0)	2 (50)	2 (50)	0.001^*^
**Education level**					
No studies	5	2 (40)	1 (20)	2 (40)	
1–6 years scholarship	23	11(47.8)	4 (17.4)	8 (34.8)	0.537
7–12 years scholarship	99	52 (52.5)	27 (27.3)	20 (20.2)	0.537
>13 years scholarship	8	6 (75)	1 (12.5)	1 (12.1)	
**Socioeconomic level**					
Low	22	11 (50)	4 (18.2)	7 (31.8)	
Middle	111	59 (53.2)	29 (26.1)	23 (20.7)	0.661
High	1	1 (100)	0 (0)	0 (0)	

* *Pearson's χ^2^-test. The results are presented in percentages and standard deviations*.

**Figure 1:**
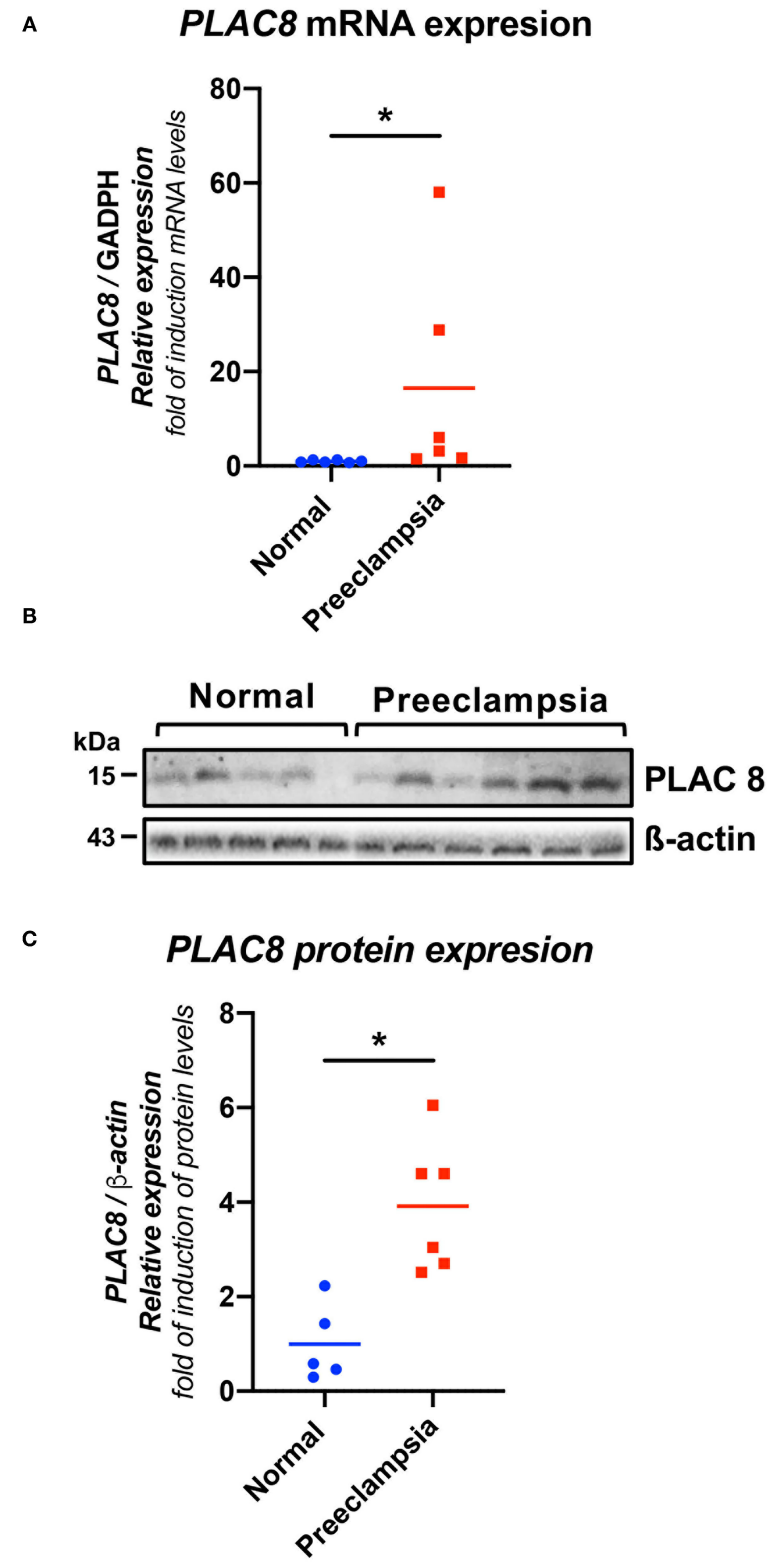
*PLAC8* gene and protein abundance in human placentas from healthy pregnancies and preeclampsia. **(A)** Real-time PCR analysis of *PLAC8* expression in normal and preeclamptic placentas. Gene expression levels were normalized to *GAPDH*. Samples: normal (*n* = 6), preeclampsia (*n* = 6). **(B)** Representative western blot for PLAC8 in protein extracts from villous tissue. **(C)** PLAC8/b-actin protein densitometries of western blotting. b-actin was used as a loading control. Samples: normal (*n* = 5), preeclampsia (*n* = 6). Data presented as means ± SD. Normal in color blue, PE represented in red Student's *t*-test. *p < 0.05.

Both the gene expression of PLAC8 in PHCT isolated from placental villi (Figure 1A) and in term placental protein expression (Figure 1B) showed significantly higher elevations in preeclampsia samples than in the HP samples (*p* < 0.05).

After direct sequencing of the five exons of the *PLAC8* gene, in a randomly selected sample of 10 cases and 10 controls, the SNV rs1143014 located in the 5′UTR was found in all cases and one control (*p* < 0.01). The evaluation of rs1143014 in the whole population revealed no significant differences in the allele and genotype frequencies between the group of cases and controls. Furthermore, the comparison after stratification showed no association with preeclampsia (OR = 0.89; CI 95%, 0.55–1.44, *p* = 0.63), obesity (OR = 0.70; CI 95%, 0.21–2.28, *p* = 0.16), or T2DM (OR = 2.88; CI 95%, 0.38–22.08, *p* = 0.39). Sequencing of the potential regulatory regions surrounding the TSS did not reveal systematic variations in either the 10 cases or 10 controls.

## Discussion

Our results suggest that pregestational obesity is associated with SPE and *PLAC8* gene, and protein expression are significantly higher in women with preeclampsia than in women with normal pregnancy.

The underlying mechanisms of the relationship between BMI and preeclampsia have not yet been clearly identified. Reduced placental perfusion, which is secondary to abnormal implantation and subsequent reduced placental vascularization, is the hallmark of preeclampsia (22). Preeclampsia is characterized by two stages. The first involves deficient placental implantation and hypoxia with the release of antiangiogenic proteins into the systemic circulation. The second, which is known as maternal syndrome, is triggered by the previous stage together with maternal predisposing factors such as obesity (23). An increase in BMI closely reflects the degree of body fat, which produces cytokines and active substances of adipose tissue (24)—all factors that are related to preeclampsia. Khadilkar et al. suggested that an increase in BMI ≥26 kg/m^2^ generates twice the risk of suffering from preeclampsia (OR = 2.1). It triples with a BMI of 30 kg/m^2^ (OR = 2.9) and increases markedly with morbid obesity (OR = 3.5) (25). Women with the lowest BMI are relatively protected against preeclampsia (24), whereas women with the highest BMI have an increased risk of SPE (26), as we also found in the present study. An epidemiological study in the southern region of Mexico revealed that the presence of overweightness is frequently observed in patients with preeclampsia (27). Authors have noticed a high frequency of preeclampsia and obesity with other comorbidities, including T2DM (28). In the present study, we found a significant association between T2DM, SPE, and MPE (OR = 2.18), which is consistent with the results of other studies that showed an increased risk of up to 3.625-fold (29). Metabolic and biochemical disturbances associated with obesity, such as chronic inflammation, oxidative stress, and decreased angiogenic factors, such as vascular endothelial growth factor (VEGF), are also features of preeclampsia (30). Clinical and experimental evidence suggests that obesity may affect placental function and perfusion through hyperlipidemia, hyperinsulinemia, or hyperleptinemia. However, the exact mechanisms are not well-understood (31). In pregnancy, the “natural” inflammatory state may promote endothelial dysfunction and ischemia, inducing the development of preeclampsia (30). Similarly, obese women have an excess of white adipose tissue and secrete a number of adipokines with putative effects on reproductive function. In this regard, leptin and adiponectin are the two most investigated (32). While adiponectin shows anti-inflammatory activity by downregulating the expression and release of proinflammatory cytokines, leptin shows proinflammatory activity. Furthermore, the hypoxic conditions prevailing in early pregnancy stages induce the placental release of proinflammatory factors such as tumor necrosis factor alpha (TNF-α) (4) and interleukin-6 (IL6), which are associated with insulin resistance and endothelial dysfunction (33). This mechanism suggests a strong relationship between obesity and preeclampsia (34).

Some nucleotide variations (SNVs) in obesity-related genes, such as *LEP* 2548G/A (35) and 668A/G in the *LPR* (36) and *LPL* (37), have been associated with preeclampsia. The *Apo E* gene is related to endothelial cell dysfunction in obesity and preeclampsia (38, 39). Furthermore, *Apo E-knockout* mice are a well-known animal model of preeclampsia that features hypertension, proteinuria, and increased expression of *sFlt-1* (40).

The *PLAC8* gene is directly involved in obesity, the placental preimplantation process, and diabetes, making it a good candidate to study in preeclampsia. In the present study, a high level of gene and protein expression of *PLAC8* at the end of pregnancy was observed in women affected by preeclampsia compared to healthy pregnant women in whom it seems to decrease as the pregnancy progresses (Figure 1). This finding coincides with the results obtained by Galaviz-Hernandez who found in a healthy murine model that the expression of *PLAC8* decreases gradually until the end of pregnancy (17). The high expression of *PLAC8* during placental implantation is directly related to low oxygen levels having an important role in trophoblast differentiation (41). However, the overexpression of the gene has been associated with early pregnancy loss in cattle (16), which suggests that a very narrow window for *PLAC8* expression is required for normal placental development, turning troublesome when high expression is abnormally maintained after such limits. The hypoxic placenta in preeclampsia maintains low oxygen levels and, as consequence, causes high *PLAC8* expression throughout pregnancy. High expression of *Plac8* is involved in adipogenesis, brown fat differentiation, and body weight control through interaction with *C/EBP*β (15), which in turn transactivates the *C/EBP* site in the *Lep* gene, stimulating its transcription (42) and its potential proinflammatory activity in preeclampsia. Thus, it is possible that sustained *PLAC8* overexpression could play a central role in the pathophysiological association between obesity and preeclampsia, maintaining the expression of leptin.

To identify whether nucleotide variations also drive the overexpression of *PLAC8*, the coding and regulatory regions of the gene were sequenced. We could not detect differences in the frequency of the observed nucleotide variations after comparing the whole studied groups. The lack of nucleotide variations associated with the changes in the expression profiles suggests the involvement of epigenetic factors induced by a hypoxic environment.

To the best of our knowledge, this is the first study investigating a potential association between the *PLAC8* gene and preeclampsia or obesity in Mexican women. These preliminary results encourage conducting basic studies to clearly identify the interrelationship between *PLAC8* and *LEPTIN* and its involvement in preeclampsia and the associated obesity.

The main limitation of the present study is the relatively small population. Consequently, larger samples and further studies in intronic regions are needed. Additionally, epigenetic mechanisms of regulation of *PLAC8* expression in preeclampsia should also be explored.

## Data Availability Statement

The original contributions presented in the study are included in the article/supplementary materials, further inquiries can be directed to the corresponding author/s.

## Ethics Statement

This study involving human participants was reviewed and approved by Ethics and Research Committee of the Ministry of Health at Durango City (Approval No. 094). The patients/participants provided their written informed consent to participate in this study.

## Author Contributions

LB-Z performed the sequencing experiments, analyzed the results, and wrote the article. LM analyzed the results. IC-W performed the western blot experiments. RB-C performed the gene expression studies. JS-P collected the samples. LS-M performed the statistical analysis. MC-R analyzed the sequencing experiments. MS-M analyzed the data and edited the manuscript. JG carried out the primary cultures of primary human cytotrophoblast, analyzed the experimental data, and edited the manuscript. CG-H designed the study, analyzed the data, and edited the manuscript. All authors contributed to the article and approved the submitted version.

## Funding

This work was supported by grants from the Secretaria de Investigación y Posgrado (SIP) of Instituto Politécnico Nacional (SIP 20211345 and SIP 20211354), the Agencia Nacional de Investigación y Desarrollo (ANID) (FONDECYT regular 1180935), and Convocatoria Nacional Subvención a la Instalación en la Academia, convocatoria año 2021, Folio No. SA77210087.

## Conflict of Interest

The authors declare that the research was conducted in the absence of any commercial or financial relationships that could be construed as a potential conflict of interest.

## Publisher's Note

All claims expressed in this article are solely those of the authors and do not necessarily represent those of their affiliated organizations, or those of the publisher, the editors and the reviewers. Any product that may be evaluated in this article, or claim that may be made by its manufacturer, is not guaranteed or endorsed by the publisher.

## Footnotes

^1^*GeneCards – The Human Gene Database*. Available online at: www.genecards.org.
